# Clozapine promotes glycolysis and myelin lipid synthesis in cultured oligodendrocytes

**DOI:** 10.3389/fncel.2014.00384

**Published:** 2014-11-18

**Authors:** Johann Steiner, Daniel Martins-de-Souza, Kolja Schiltz, Zoltan Sarnyai, Sabine Westphal, Berend Isermann, Henrik Dobrowolny, Christoph W. Turck, Bernhard Bogerts, Hans-Gert Bernstein, Tamas L. Horvath, Lorenz Schild, Gerburg Keilhoff

**Affiliations:** ^1^Department of Psychiatry and Psychotherapy, University of MagdeburgMagdeburg, Germany; ^2^Center for Behavioral Brain SciencesMagdeburg, Germany; ^3^Pembroke College, University of CambridgeCambridge, UK; ^4^Department of Translational Research in Psychiatry, Max Planck Institute of PsychiatryMunich, Germany; ^5^Laboratory of Neuroproteomics, Department of Biochemistry and Tissue Biology, Institute of Biology, University of Campinas (UNICAMP)Campinas, Brazil; ^6^Laboratory of Psychiatric Neuroscience, James Cook UniversityTownsville, QLD, Australia; ^7^Comparative Genome Centre, James Cook UniversityTownsville, QLD, Australia; ^8^Centre for Biodiscovery and Molecular Development of Therapeutics, James Cook UniversityTownsville, QLD, Australia; ^9^Australian Institute of Tropical Health and Medicine, James Cook UniversityTownsville, QLD, Australia; ^10^Institute of Clinical Chemistry and Pathobiochemistry, University of MagdeburgMagdeburg, Germany; ^11^Section of Comparative Medicine, Yale University School of MedicineNew Haven, CT, USA; ^12^Institute of Biochemistry and Cell Biology, University of MagdeburgMagdeburg, Germany

**Keywords:** schizophrenia, oligodendrocytes, clozapine, haloperidol, glycolysis, myelin

## Abstract

Clozapine displays stronger systemic metabolic side effects than haloperidol and it has been hypothesized that therapeutic antipsychotic and adverse metabolic effects of these drugs are related. Considering that cerebral disconnectivity through oligodendrocyte dysfunction has been implicated in schizophrenia, it is important to determine the effect of these drugs on oligodendrocyte energy metabolism and myelin lipid production. Effects of clozapine and haloperidol on glucose and myelin lipid metabolism were evaluated and compared in cultured OLN-93 oligodendrocytes. First, glycolytic activity was assessed by measurement of extra- and intracellular glucose and lactate levels. Next, the expression of glucose (GLUT) and monocarboxylate (MCT) transporters was determined after 6 and 24 h. And finally mitochondrial respiration, acetyl-CoA carboxylase, free fatty acids, and expression of the myelin lipid galactocerebroside were analyzed. Both drugs altered oligodendrocyte glucose metabolism, but in opposite directions. Clozapine improved the glucose uptake, production and release of lactate, without altering GLUT and MCT. In contrast, haloperidol led to higher extracellular levels of glucose and lower levels of lactate, suggesting reduced glycolysis. Antipsychotics did not alter significantly the number of functionally intact mitochondria, but clozapine enhanced the efficacy of oxidative phosphorylation and expression of galactocerebroside. Our findings support the superior impact of clozapine on white matter integrity in schizophrenia as previously observed, suggesting that this drug improves the energy supply and myelin lipid synthesis in oligodendrocytes. Characterizing the underlying signal transduction pathways may pave the way for novel oligodendrocyte-directed schizophrenia therapies.

## Introduction

Schizophrenia is a devastating mental disorder affecting about 1% of the population worldwide (Saha et al., 2005). The current pharmacological treatment is only partially effective and induces numerous side-effects, leading to non-compliance or long-term health consequences (Newcomer, 2007). The relative lack of progress in developing better drugs to treat the disease is partly due to incomplete understanding of disease pathophysiology and the mechanisms of drug action.

Fundamental neuroscience research demonstrates that the brain is one of the most energy demanding tissues in the body and is exquisitely sensitive to perturbations of energy metabolism (Magistretti and Allaman, 2013). Therefore, absolute or relative energy insufficiency due to abnormal glucose metabolism can lead to abnormal behavior and cognition. The link between schizophrenia and abnormal glucose metabolism was first reported well-before the introduction of antipsychotics by Maudsley in the late 19th century who showed that diabetes occurred more frequently in families with history of “insanity” (Mukherjee et al., 1989). Furthermore, when applying insulin therapy in patients with psychiatric disorders, psychotic patients required higher doses of insulin compared to non-psychotic subjects, indicating some degree of insulin resistance (Sakel, 1938). Several recent studies show elevated rates of either diabetes or impaired glucose tolerance (insulin) resistance in first-episode, drug-naive subjects and in non-psychotic relatives of patients, suggesting that altered glucose metabolism might be related to schizophrenia itself, rather than only to treatment, or lifestyle factors related to it (Kirkpatrick et al., 2012; Van Welie et al., 2013).

Elevated glucose levels and reduced lactate levels were identified in the cerebrospinal fluid of first onset drug-naïve schizophrenia patients (Holmes et al., 2006). Interestingly, short-term treatment with atypical antipsychotic drugs (prototype drug: clozapine) resulted in a normalization of the cerebrospinal fluid metabolite profile in approximately 50% of patients, whereas typical antipsychotics (prototype drug: haloperidol) did not show such an effect (Holmes et al., 2006). In post-mortem brain studies, decreased expression of glycolytic and glycogen synthesis enzymes and increased expression of glycogenolytic enzymes were found (Prabakaran et al., 2004). More recently, increased circulating levels of insulin and insulin-related peptides have been identified in independent cohorts of first-onset, drug-naïve schizophrenics even though blood glucose levels were relatively normal, suggesting insulin resistance as a disease-inherent factor (Guest et al., 2010). Furthermore, reduced glucose utilization has been found in different brain regions of schizophrenics by neuroimaging studies (Buchsbaum and Hazlett, 1998; Buchsbaum et al., 2007). In addition, genetic studies have identified linkages between genes involved in glucose metabolism with an elevated risk for schizophrenia (Stone et al., 2004).

**Figure 1:**
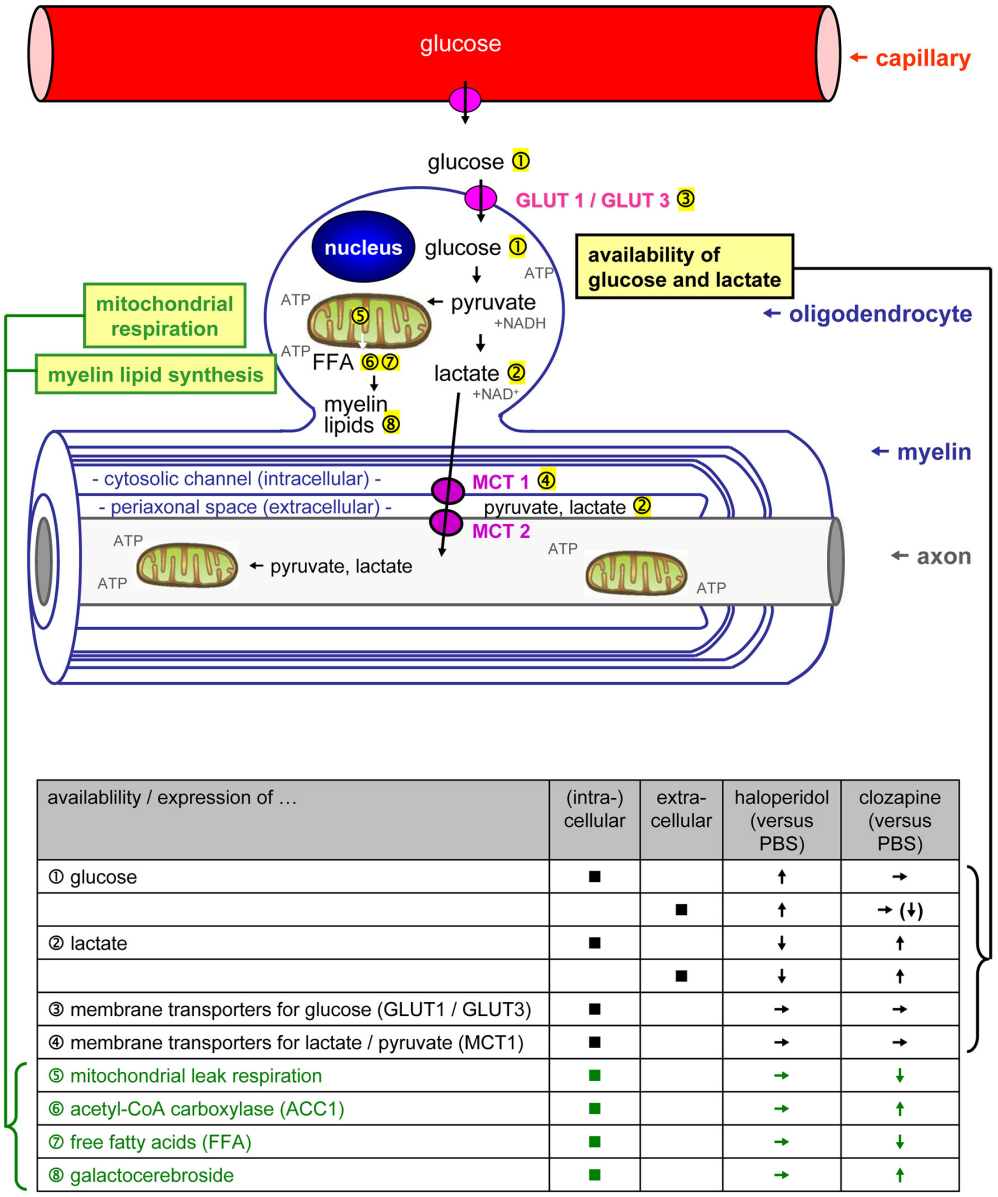
**The scheme is showing *previously described mechanisms* how glycolytic oligodendrocytes maintain myelin and axonal integrity [adapted from Fünfschilling et al. (2012) and Lee et al. (2012) by permission of Macmillian Publishers Ltd]**. Note that myelinated axons are separated by a thin periaxonal space (extracellular) from the oligodendroglial cytoplasm filling the inner loops of myelin (cytosolic channel/intracellular). The table below summarizes *our present study results* on the differential regulation of oligodendrocytic glucose and lactate homeostasis (black text and symbols), as well as myelin lipid synthesis (green text and symbols) by haloperidol and clozapine. *Abbreviations:* ATP, adenosine triphosphate; free fatty acids FFA, free fatty acids; GLUT, glucose transporter; MCT, monocarboxylate transporter; NAD^+^/NADH, nicotine amide-adenine-dinucleotide.

Oligodendrocytes are involved in maintaining myelin integrity, rapid saltatory conduction, and functional connectivity between distant brain areas, and it has been postulated that they are crucial for maintaining axonal energy supply and myelin integrity (Bernstein et al., 2009; Schmitt et al., 2009). Recent studies showed that oligodendrocytes can import glucose from the extracellular space and from astrocytes to drive glycolysis and the tricarboxylic acid cycle (Fünfschilling et al., 2012; Lee et al., 2012). The end products of glycolysis are lactate or pyruvate which can be directly transferred from oligodendrocytes to myelinated axons via monocarboxylate transporters (MCT 1/2) (Figure 1, Fünfschilling et al., 2012; Lee et al., 2012). Additionally, glucose and lactate foster the synthesis of free fatty acids and myelin lipids.

Oligodendrocyte loss or dysfunction and abnormal metabolic activity have been identified in schizophrenia (Tkachev et al., 2003; Uranova et al., 2004; Haroutunian et al., 2007; Schmitt et al., 2009; Martins-De-Souza et al., 2011a; Bernstein et al., 2014). In addition, levels of the myelin-forming phospholipids phosphatidylcholine, sphingomyelin, and galactocerebroside were decreased in the thalamus of schizophrenia patients treated with *typical* antipsychotics, such as haloperidol (Schmitt et al., 2004). We have shown that haloperidol and clozapine attenuate glucose-deprivation induced necrotic cell death in oligodendrocyte culture, suggesting that antipsychotic drugs may exert a protective effect on oligoendrocytes during a glucose/energy deprived state (Steiner et al., 2011).

Clozapine has stronger systemic metabolic side effects than haloperidol. We hypothesized that the therapeutic antipsychotic and adverse metabolic effects might be related. Considering that cerebral disconnectivity through oligodendrocyte dysfunction has been implicated in schizophrenia, it is important to determine the effect of atypical/typical prototype drugs on oligodendrocyte energy metabolism and myelin lipid production. To test this, we have used OLN-93 oligodendrocyte cells which express a number of oligodendrocyte markers/neurotransmitter receptors (e.g., NG2, CNP, MAG, MOG, Olig1, Olig2, PLP, 5HT1A, 5HT2A, D2, D3, D4, M4) and assessed the metabolic responses after exposure to either clozapine or haloperidol which is known to cause fewer peripheral metabolic side effects. Glucose and lactate homeostasis were measured by determining intra- and extracellular glucose and lactate levels as well as the expression levels of glucose (GLUT) and monocarboxylate (MCT) transporters (Bell et al., 1990; Pierre et al., 2007; Merezhinskaya and Fishbein, 2009). For assessing the effects of these drugs on myelin synthesis, we measured acetyl-CoA carboxylase (ACC1), free fatty acids (FFA), and galactocerebroside^1^.

## Methods and materials

### OLN-93 cell culture

Oligodendroglial OLN-93 cultures were kept as previously described (Steiner et al., 2010, 2011; Mosebach et al., 2013). After 6 or 24 h, media and cell homogenates were collected and stored at −80°C until further analysis (Steiner et al., 2010, 2011; Mosebach et al., 2013).

### Availability of lactate and glucose

The levels of haloperidol and clozapine in the brain tissue are 10- to 30-fold higher than the therapeutic plasma concentrations (haloperidol: 5–20 ng/mL; clozapine: 100–600 ng/mL) (Zhang et al., 2007). Therefore, the effects of antipsychotic medication on the energy metabolism of OLN-93 cells were analyzed by adding a vehicle (0.01% HCl, same dissolving solution used to solubilize the antipsychotics), 0.1 or 1 μg/ml of haloperidol and 1 or 10 μg/ml of clozapine (Sigma-Aldrich, Taufkirchen, Germany) to the cell culture for 6 or 24 h (Steiner et al., 2011).

#### Intra- and extracellular lactate and glucose concentrations (Figure 1①–②)

Glucose and lactate concentrations were determined in cell homogenates and media by commercial assays (Modular Analytics System™, Roche Diagnostics, Mannheim, Germany). Three of the 15 dishes per experimental setting were pooled for these analyses.

#### Membrane transporters for lactate and glucose (Figure 1③–④)

The expression of MCT1-4 and GLUT1-4 was tested in OLN-93 cells by reverse transcriptase polymerase chain reaction (RT-PCR). Five of the 15 dishes per experimental setting were pooled for these analyses.

Total ribonucleic acid (RNA) was isolated from OLN-93 cell cultures using guanidinium isothiocyanate/phenol/chloroform (peqGOLD TriFast™, peqlab, Erlangen, Germany). For removing deoxyribonucleic acid (DNA) contamination, 5 μg of the total cell RNA was treated with Turbo DNA-free (Ambion, Austin, TX, USA) according to the manufacturer's instructions. RNA (4.5 μl; 2.25 μg input RNA) was reverse transcribed using the RevertAid™ H Minus First strand cDNA Synthesis Kit primed with Oligo(dT)_18_ primers (Fermentas, St. Leon-Rot, Germany; primers listed in the Table 1). cDNA (2 μL) was then PCR-amplified with Taq-DNA-polymerase (peqlab, Erlangen, Germany).

**Table 1:** **Primer sequences for reverse transcriptase polymerase chain reaction (RT-PCR) analyses**.

**Gene**	**Sequence (5′→**3**′)**	**GenBank accession number**	**Product size, bp**
*rMCT1_f*	AGAAGTCAGCCTTCCTCCTTT (21)	D63834	394
	CCACAAGCCCAGTATGTGTAT (21)		
*rMCT2_f*	GGCCTTCGGTAGGATTAATAG (21)	X97445	367
	ATGCCTGATGATAACACGACT (21)		
*rMCT3_f*	GCTCTGAAGAACTATGAAATCA (22)	AF059258	427
	GTGAACAGGGTCTAACATATTG (22)		
*rMCT4_f*	TGCGGCCCTACTCTGTCTAC (20)	U87627	369
	TCTTCCCGATGCAGAAGAAG (20)		
*rGLUT1_f*	GCCTGAGACCAGTTGAAAGCAC (22)	S68135	292
	CTGCTTAGGTAAAGTTACAGGAG (23)		
*rGLUT2_f*	TTGGCTTTCACTGTCTTCACT (21)	J03145	811
	CTTCCTTTTCTTTCCTCATCTC (22)		
*rGLUT3_f*	AACAGAAAGGAGGAAGACCA (20)	U17978	630
	CGCAGCCGAGGGGAAGAACA (20)		
*rGLUT4_f*	AGTGCCTGAGTCTTCTTT (18)	J04524	486
	TGATGTTAGCCCTGAGTAG (19)		
*GAPDH_f*	TTAGCACCCCTGGCCAAGG (19)	XM228411	531
	CTTACTCCTTGGAGGCCATG (20)		

*Annotation: Annealing temperature for all primers: 55°C; cycle number (within linear range) for all MCTs and GLUTs: 34, for GAPDH: 24*.

One-tenth of each reaction product was electrophoresed on a 1% agarose gel. The PCR product bands were quantified by densitometric analysis using a Biometra BioDocAnalyzer. Using glyceraldehyde-3-phosphate dehydrogenase (GAPDH) as a housekeeping gene for data normalization (GAPDH showed no medication-induced changes in expression), the ratio of MCT and GLUT expression to GAPDH expression was calculated.

### Mitochondrial respiration (Figure 1⑤)

Oxygen consumption was assessed in OLN-93 cell suspensions with a Clark-type electrode in a temperature regulated incubation chamber (high-resolution oxygraph™, Paar Physica, Vienna, Austria) at 6 and 24 h after the addition of PBS, 1 μg/ml of haloperidol or 10 μg/ml of clozapine. Three of the 15 dishes per experimental setting were pooled for these analyses. The oxygen content of the air-saturated medium was 435 ng atoms O/ml at 30°C (Steiner et al., 2010). To analyze mitochondrial energy metabolism in OLN-93 oligodendrocytes, cells were scraped from dishes and put with 2 ml of growth medium into the incubation chamber of the oxygraph. Basal respiration was assessed to characterize the actual activity of oxidative phosphorylation. Then, mitochondrial ATP synthesis was blocked by adding 5 μM oligomycin (oli, Sigma-Aldrich, Taufkirchen, Germany). The remaining oxygen consumption reflects the proton leak of the mitochondrial membrane system. Finally, maximal respiration was stimulated by the addition of 5 μM of the uncoupler p-trifluoromethoxyphenylhydrazone (FCCP, Sigma-Aldrich, Taufkirchen, Germany). This oxygen consumption reflects the capacity of the respiratory chain, while the FCCP/protein ratio reflects the cellular content of functionally intact mitochondria. The FCCP/oli ratio was calculated as an estimate of the capacity of the respiratory chain to support oxidative phosphorylation.

### Myelin lipid synthesis

#### Acetyl-Coa carboxylase (ACC1) (Figure 1⑥)

For Western blotting, proteins were extracted as described previously (Martins-De-Souza et al., 2011b). Five of the 15 dishes per experimental setting were pooled for these analyses. Fifty μg of total protein from each control and haloperidol- and clozapine treated samples were run on a 12% SDS minigel (BioRad, Hercules, CA, USA). Proteins were transferred to Immobilon PVDF membranes (Millipore, Bedford, MA, USA) at 100 V for 1 h using a cooling system. Membranes were treated with 5% carnation instant nonfat dry milk in Tris-Buffered Saline containing 0.1% Tween 20 (TBS-T) for 4 h and rinsed in TBS-T three times for 20 min. Next, the membranes were incubated with rabbit ACC1 antibody (Abcam, Cambridge, UK) at a 1:1000 dilution in TBS-T overnight at 4°C. After the incubation, membranes were washed 3 times with TBS-T for 15 min per wash and incubated with anti-rabbit IgG horseradish peroxidase conjugate (GEHealthcare, Uppsala, Sweden) for 40 min at room temperature. The membranes were subjected to a final wash with water and TBS-T, incubated with ECL mixture (GE Healthcare) for 1 min and scanned in a ChemiDoc™ System (BioRad). Band signals (optical densities) were assessed using Quantity One™ software (BioRad).

#### Free fatty acids (Figure 1⑦)

FFA levels were measured in the homogenates using the Free fatty acid quantification kit™ (BioVision, Mountain View, CA, USA), following the manufacturer's instructions. Five of the 15 dishes per experimental setting were pooled for these analyses.

#### Galactocerebroside (Figure 1⑧)

Galactocerebroside levels were measured in order to assess the extent of myelination (6 and 24 h after the addition of phosphate-buffered saline (PBS) (pH 7.4), 1 μg/ml haloperidol or 10 μg/ml clozapine). Six dishes were used per experimental setting.

For the immunostaining, cell cultures were washed twice with, fixed for 30 min in 4% PBS-buffered paraformaldehyde, and incubated at room temperature for 3 h with a 1:1000 dilution of the monoclonal mouse anti-galactocerebroside antibody (MAB342; Chemicon, Temecula, CA, USA) (Steiner et al., 2010, 2011). Cells were washed three times for 5 min with PBS and incubated with the respective secondary antibody (Molecular Probes, Göttingen, Germany) at a 1:500 dilution: Alexa Fluor 488 (A11055; goat anti-mouse-IgG; green fluorescence).

Three randomly chosen fields of vision from each of the six dishes per experimental setting were scanned using an AxioImager (Zeiss, Jena, Germany) with a Plan-Neofluar objective (x 40/0.75). The overall galactocerebroside immunostaining intensity of each image was measured in a standard evaluation window (500 × 300 pixels) using the ImageJ software (http://rsbweb.nih.gov/ij/). The mean of each dish was calculated.

### Statistical analysis

Cell culture data were normally distributed, as indicated by Kolmogorov–Smirnov tests. Thus, analyses of variance (ANOVA) were applied in order to compare the influence of treatment conditions on the concentrations of glucose or lactate in cell homogenates or supernatants. Dunnett's test was applied for *post-hoc* comparisons. Significance was defined as *P* < 0.05, while a probability level of 0.05 ≤ *P* < 0.10 was considered as a statistical trend.

## Results

### Availability of lactate and glucose

#### Intra- and extracellular lactate and glucose concentrations (Figure 1①–②)

**Figure 2:**
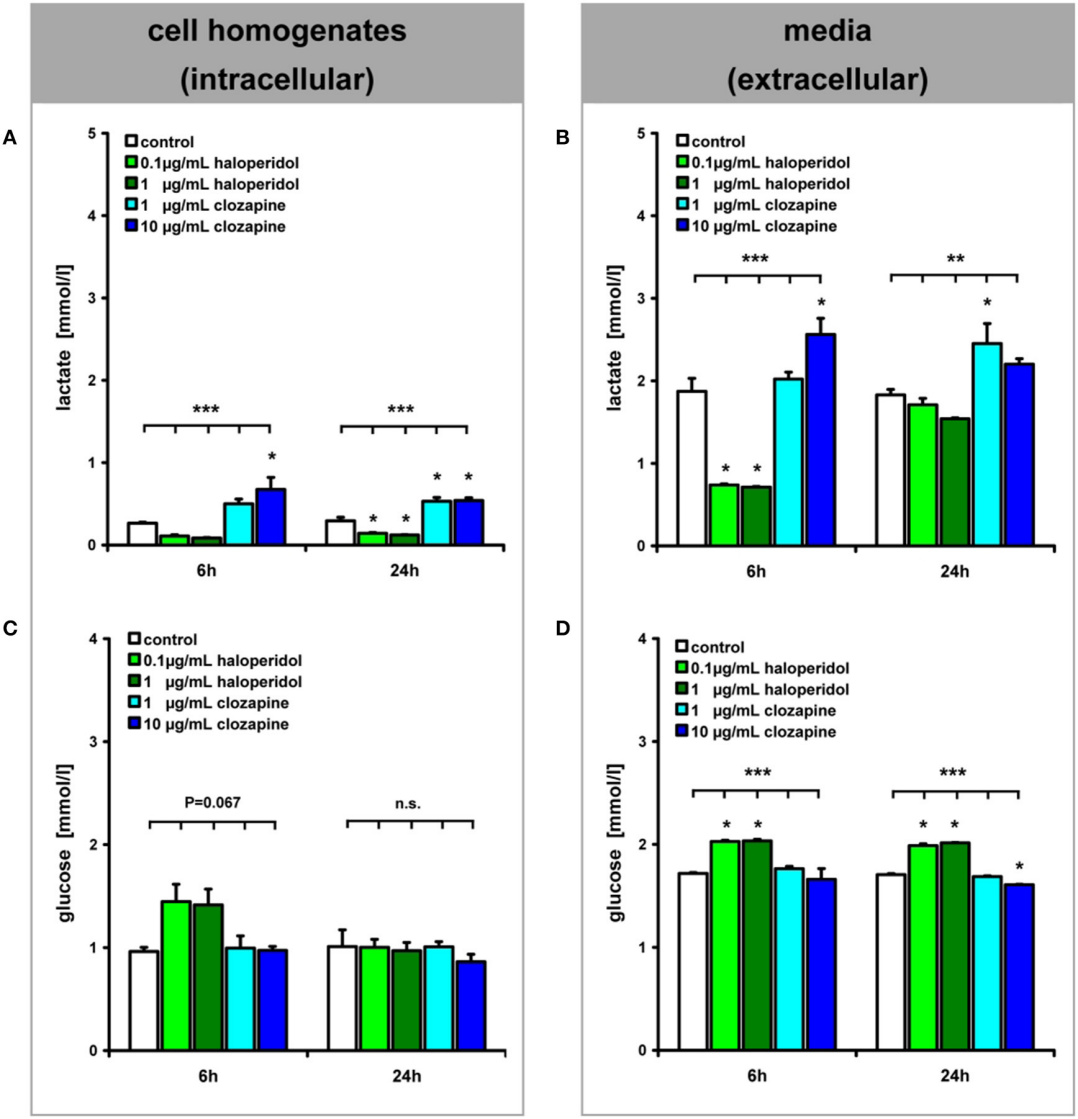
**(see Figure 1 ①–②): Lactate and glucose concentrations in OLN-93 cell homogenates (A,C) and media (B,D) are shown after 6 and 24 h of treatment**. *Annotation:* Data are given as mean ± s.e.m. ^**^P < 0.01, ^***^P < 0.001, n.s. not significant; three of the 15 dishes per experimental setting were pooled for these analyses.

Addition of clozapine and haloperidol to OLN-93 cells had different effects on lactate and glucose levels. In clozapine treated cells, lactate levels were increased, while the opposite effect was observed in cells exposed to haloperidol (Figures 2A,B). For both drugs, this effect was evident after 6 h in the extracellular medium [main effect (TREAT) *F*_(4, 18)_ = 46.25, *P* < 0.001, low clozapine (LC) n.s., high clozapine (HC) *P* < 0.01, low haloperidol (LH) *P* < 0.001, high haloperidol (HH) *P* < 0.001] and within cells [TREAT *F*_(4, 18)_ = 11.30, *P* < 0.001, LC n.s., HC *P* < 0.01, LH n.s., HH n.s.]. This effect persisted after 24 h of incubation in the extracellular medium [TREAT *F*_(4, 20)_ = 7.21, *P* < 0.01, LC *P* < 0.05, HC, LH, and HH n.s.] and in cells [TREAT *F*_(4, 20)_ = 42.07, *P* < 0.001, LC *P* < 0.001, HC *P* < 0.001, LH *P* < 0.01, HH *P* < 0.01]. The effect of clozapine did not appear to be sensitive to concentration (Three-Way ANOVA with factors concentration, compartment, time; main effect of concentration: *F*_(1,3)_ = 0.32, n.s.] while a concentration effect was evident for haloperidol [*F*_(1,4)_ = 10.72, *P* < 0.05]. Also, the effect of haloperidol changed across time [*F*_(1,4)_ = 720,01, *P* < 0.001] for the extra- and intracellular compartments [compartment × time interaction, *F*_(1,4)_ = 324,84, *P* < 0.001], reflecting the return of lactate to normal levels after 24 h and suppression inside the cells.

The glucose concentrations showed an inverse pattern of drug effects (Figures 2C,D). Haloperidol treatment led to increased glucose concentrations in the extracellular medium after 6 h of incubation [TREAT *F*_(4,18)_ = 20.99, *P* < 0.001, LC and HC n.s., LH *P* < 0.001, HH *P* < 0.001] which was also evident within cells but this was not statistically significant [TREAT *F*_(4, 18)_ = 2.66, *P* = 0.067, LH *P* = 0.051, HC, LC, and HH n.s.]. After 24 h this effect was observed at the extracellular level [TREAT *F*_(4, 20)_ = 360.66, *P* < 0.001, LC n.s., HC *P* < 0.001, LH *P* < 0.001, HH *P* < 0.001]. At the same time, haloperidol also increased glucose levels but, in contrast, 10 μg/mL of clozapine led to a decrease in glucose levels in the medium, possibly reflecting increased glucose turnover.

#### Membrane transporters for glucose and lactate (Figure 1③–④)

**Figure 3:**
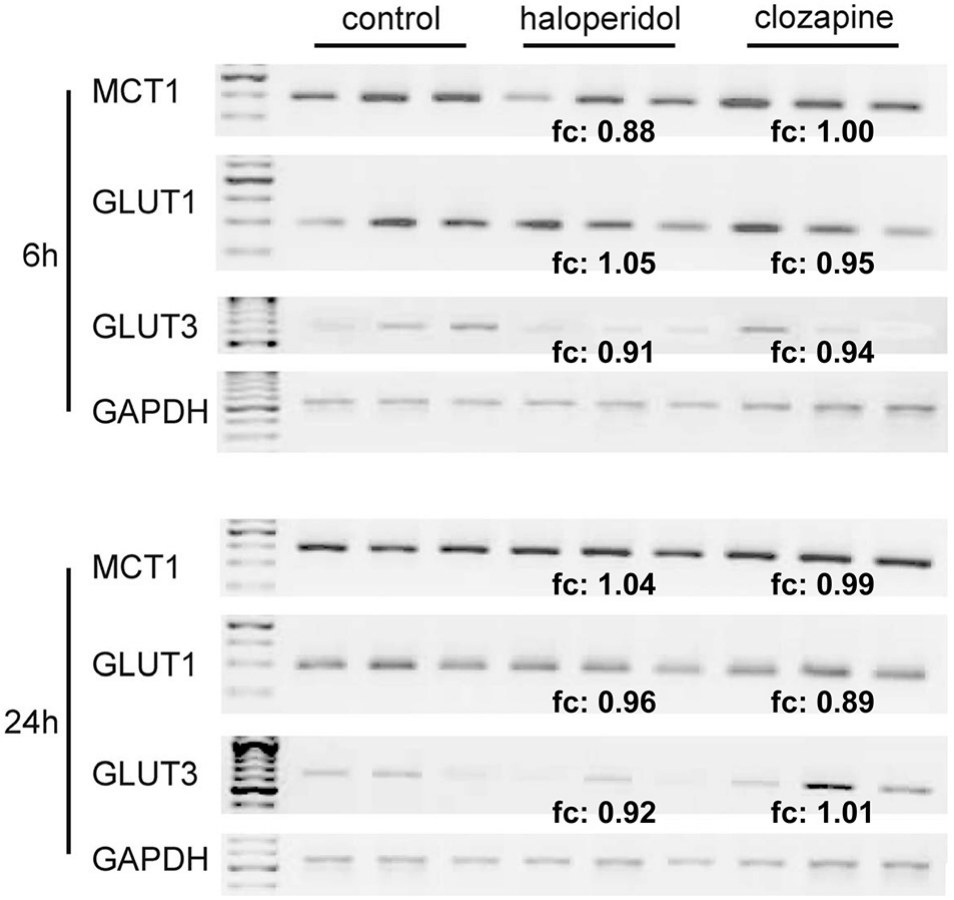
**(see Figure 1 ③–④): RT-PCR analysis of MCT1-, GLUT1- and GLUT3-expression in relation to the housekeeping gene GAPDH in OLN-93 cells**. Compared to the control condition, 1 μg/ml of haloperidol and 10 μg/ml of clozapine had no significant effect on the expression of these transporters. MCT2, MCT3, MCT4, and GLUT4 were not expressed in OLN-93 cells. *Annotation:* Results from three tests per treatment condition are presented (five of the 15 dishes per experimental setting were pooled for the RT-PCR analyses). *Abbreviations:* fc, fold change, ratio describing how much the expression of respective membrane transporters (compared to GAPDH expression) changes from the control condition to haloperidol or clozapine treatment; GAPDH, glyceraldehyde-3-phosphate dehydrogenase, one of the most commonly used housekeeping genes used in comparisons of gene expression data; GLUT, glucose transporter, MCT, monocarboxylate transporter. Annealing temperature for all primers: 55°C; cycle number (within linear range) for all MCTs and GLUTs: 34, for GAPDH: 24.

RT-PCR analyses revealed the expression of MCT1, GLUT1, and GLUT3 but not the expression of MCT2, MCT3, MCT4, or GLUT4 in OLN-93 cells (Figure 3). Neither incubation with haloperidol (1 μg/ml) or clozapine (10 μg/ml) had an effect on the expression of these transporters in comparison to the basal condition.

### Mitochondrial respiration (figure 1⑤)

No significant differences were observed between the FCCP/protein ratio in PBS-treated cells and either haloperidol- or clozapine-treated cells after 6 or 24 h (Table 2). These data indicate that the cellular content of functionally intact mitochondria was not significantly affected by the administration of haloperidol or clozapine to OLN93 cultures. However, clozapine treatment led to a significant increase in the ratio between FCCP stimulated respiration and the respiration in the presence of the ATP-synthase inhibitor oligomycin. Thus, clozapine apparently lowers oxygen consumption coupled to passive proton leakage through the mitochondrial membrane system and thereby increases the capacity of the respiratory chain for ATP synthesis.

**Table 2:** **(see Figure 1): Mitochondrial respiration in terms of oxygen consumption was assessed with a Clark-type electrode in a temperature regulated incubation chamber (Reynafarje et al., 1985)**.

**Time (h)**	**Parameter**	**PBS**	**Haloperidol**	**Clozapine**	***P*-value haloperidol vs. PBS**	***P*-value clozapine vs. PBS**
6	FCCP/protein (ng atom O/min/mg)	63.16 ± 13.57	77.78 ± 26.10	71.51 ± 13.05	0.373	0.701
6	FCCP/oli	9.20 ± 1.10	13.98 ± 3.40	13.77 ± 1.94	0.193	0.116
24	FCCP/protein (ng atom O/min/mg)	69.95 ± 19.84	66.82 ± 6.79	79.34 ± 10.96	0.892	0.479
24	FCCP/oli	**4.70±1.66**	8.37 ± 3.99	**10.77±3.68**	0.224	0.039^*^

*Annotations: FCCP, cellular respiration after the addition of p-trifluoromethoxyphenylhydrazone, a protonophore and uncoupler of mitochondrial oxidative phosphorylation; the FCCP/protein ratio is an estimate of the cellular content of functionally intact mitochondria in the analyzed OLN-93 cells; oli, cellular respiration after the addition of oligomycin, an inhibitor of mitochondrial adenosine triphosphatase; the FCCP/oli ratio is an estimate of the efficacy of mitochondrial respiration. Data are given as mean ± SD from n = 5 cultures per experimental setting*;

* *P < 0.05*.

### Myelin lipid synthesis

#### Acetyl-Coa carboxylase (ACC1) (Figure 1⑥)

**Figure 4:**
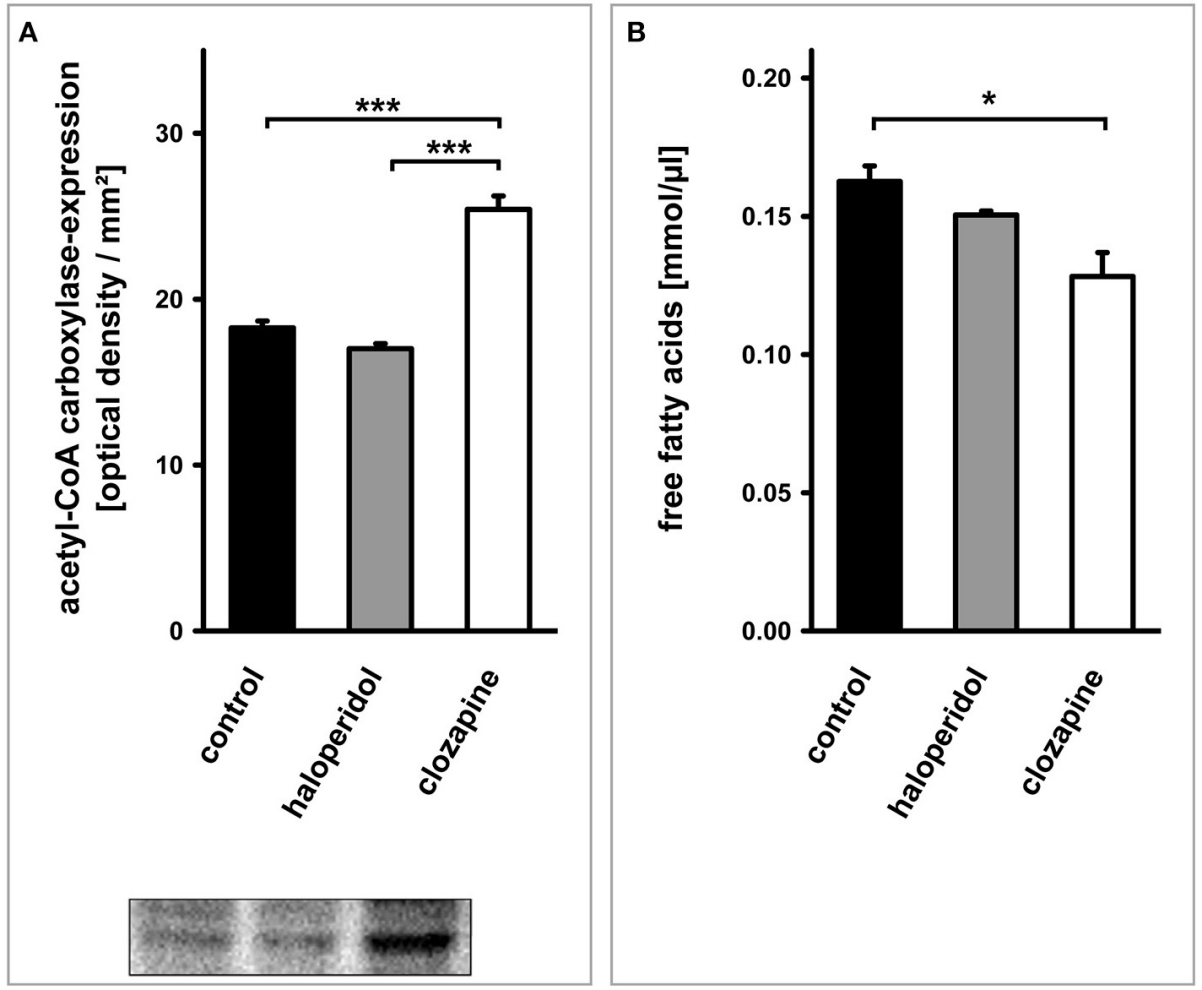
**(see Figure 1 ⑥–⑦): (A)** Western blot analysis of acetyl-CoA carboxylase (ACC1)-expression in OLN-93 cells. Antipsychotic treatment with clozapine led to a significant increase in the expression of this rate limiting enzyme for fatty acid synthesis compared to the control condition or haloperidol treatment. **(B)** The cellular concentration of free fatty acids was dependent on the treatment condition. Clozapine led to a significant decrease in the free fatty acid content of OLN-93 cells. *Annotation:* Data are given as mean ± s.e.m. ^*^P < 0.05, ^***^P < 0.001.

The expression of ACC1 in OLN-93 cells after 24 h differed significantly between clozapine and haloperidol treatment as well as the control condition [Figure 4A; TREAT *F*_(2,6)_ = 68.17, *P* < 0.001]. Treatment with clozapine was associated with an increased expression of ACC1 (clozapine vs. control condition: *P* < 0.001; clozapine vs. haloperidol: *P* < 0.001).

#### Free fatty acids (Figure 1⑦)

The cellular concentration of FFA after 24 h differed significantly between treatments [Figure 4B; TREAT *F*_(2,6)_ = 8.42, *P* < 0.05]. Again, this significant difference was due to a clozapine effect which led to a reduced cellular concentration of FFA (clozapine vs. control condition: *P* < 0.05; clozapine vs. haloperidol: *P* = 0.069).

#### Galactocerebroside (Figure 1⑧)

**Figure 5:**
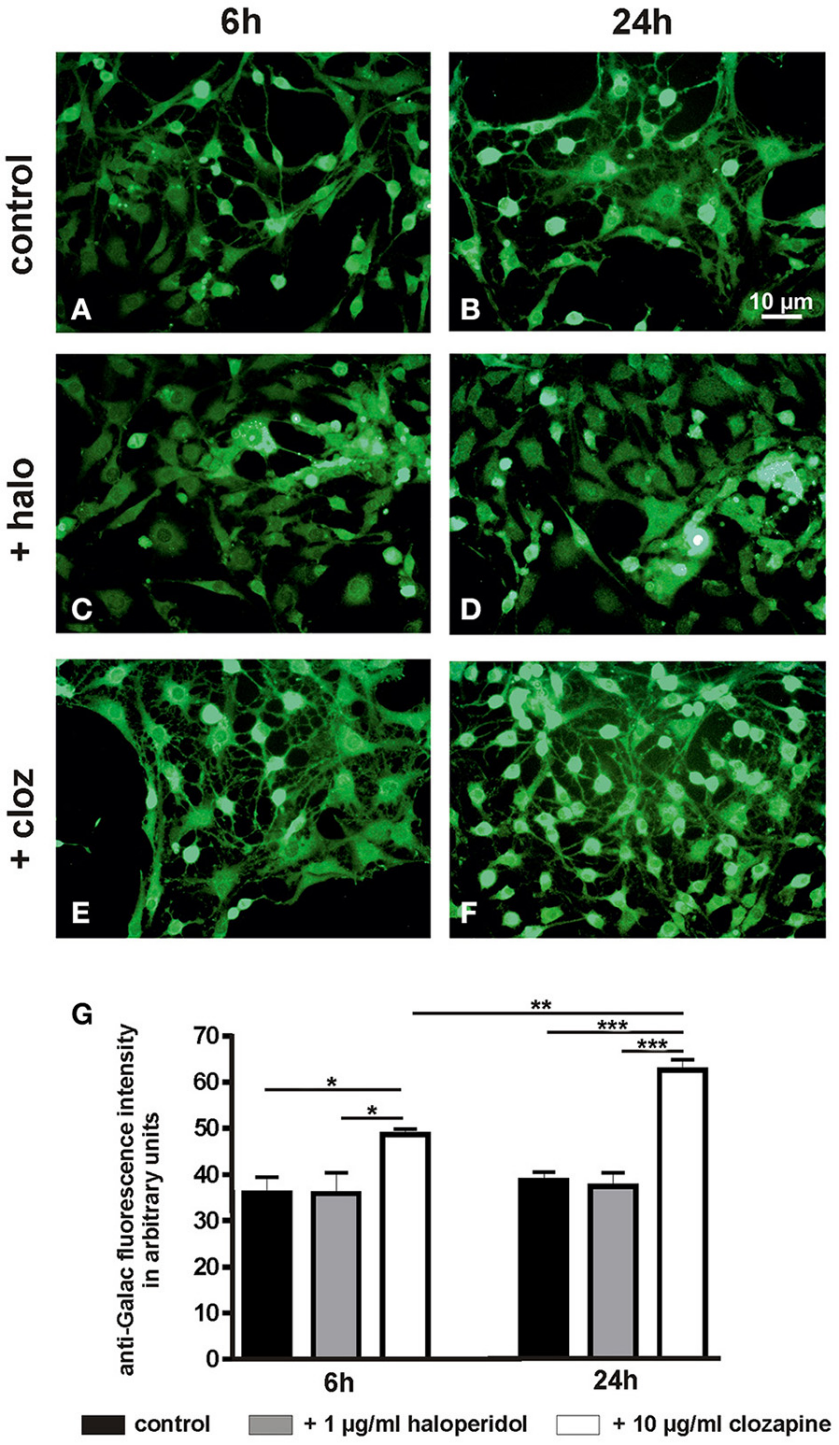
**(see Figure 1): (A–F)** Photographs display examples of the galactocerebroside immunostaining in control cultures and after treatment with haloperidol (+halo) or clozapine (+cloz) for 6 and 24 h. **(G)** Quantitative evaluation of the galactocerebroside immunostaining intensity revealed a higher expression of this characteristic myelin lipid in clozapine-treated OLN-93 oligodendrocytes. Notably, clozapine-treated cells showed also a more mature cytomorphology. *Annotation:* Data are given as mean ± s.e.m. from *n* = 6 cultures per experimental setting; ^*^P < 0.05, ^**^P < 0.01, ^***^P < 0.001. The scale bar in Figure 5B is representative for all photographs in Figure 5.

Quantitative evaluation (Figure 5) revealed a dependency of galactocerebroside expression on the treatment condition [6 h: TREAT *F*_(2, 15)_ = 4.41, *P* < 0.05; 24 h: TREAT *F*_(2, 15)_ = 35.11, *P* < 0.001]. This was caused by a higher level of this characteristic myelin lipid in clozapine-treated OLN-93 oligodendrocytes after 6 h (clozapine vs. control: *P* < 0.05, clozapine vs. haloperidol: *P* < 0.05) and 24 h (clozapine vs. control: *P* < 0.001, clozapine vs. haloperidol: *P* < 0.001). Clozapine treatment led to a significant increase in galactocerebroside expression during the time course from 6 to 24 h [*F*_(1, 10)_ = 24.63, *P* < 0.01], while this effect was absent in haloperidol treated cultures [*F*_(1, 10)_ = 0.09, *P* = 0.77].

## Discussion

This study is the first to present a comparative analysis of the effects of clozapine and haloperidol on metabolism in oligodendrocytes that are known to be crucial in maintaining brain connectivity. Consistent with the hypothesis that these two prototypical first and second generation antipsychotic drugs might exert their well-known differential therapeutic effects due to differential modulation of oligodendrocyte metabolism, this study experimentally elaborates on their respective impact. The results yield clear cut differences between clozapine and haloperidol. Clozapine promotes glucose utilization by oligodendrocytes as reflected by decreased glucose and increased lactate which is crucial for neuronal energy supply. Moreover, clozapine fosters mitochondrial respiration, thereby promoting cellular energy production and lipid synthesis supporting myelination and thus neuronal connectivity. In contrast, haloperidol appeared to inhibit glucose utilization by the oligodendrocytes and led to increased consumption of lactate as reflected by decreased lactate abundance after incubation.

### Availability of glucose and lactate (Figures 1 ①–④, 2)

Haloperidol treatment decreased glycolysis in OLN-93 oligodendrocytes, as shown by increased intracellular and extracellular glucose levels, and lactate levels were reduced. Increased levels of glucose in the media suggest that the haloperidol-treated OLN93 cells consumed glucose at a lower rate than untreated cells. Conversely, clozapine treatment led to decreased extracellular glucose levels and increased intra- and extracellular lactate levels within 24 h, indicating increased glycolysis compared to untreated cells. The finding that lactate is released from clozapine-treated OLN-93 cells suggests that oligodendrocytes treated with clozapine *in vivo* also provide increased amounts of lactate, which may result in additional energy for axons (Fünfschilling et al., 2012; Lee et al., 2012). Axons are known to express MCT2, an important transport protein for the uptake of lactate (Pierre et al., 2007; Merezhinskaya and Fishbein, 2009).

Surplus glucose has been added to the culture medium. Thus, it is unlikely that the observed effects were caused by impaired glucose supply. We interpret the presented results rather as a consequence of changes in the turnover of glucose because of an altered demand of glucose. In the case of clozapine, glucose turnover seems to be increased due to increased lipid production [increased ACC1 and galactocerebroside expression; see Myelin Lipid Synthesis (Figures 1 ⑥–⑧, 4, 5)]. An interpretation of the observed haloperidol effects on glucose and lactate levels is less clear since the demand of glucose seems to be reduced while we did not find a significantly reduced lipid synthesis.

Haloperidol and clozapine had no significant influence on the expression of MCT1, GLUT1, or GLUT3 in OLN-93 cells (Figure 3). The observed expression pattern of these transporters in OLN-93 oligodendrocytes is in line with the literature (Pierre et al., 2007; Merezhinskaya and Fishbein, 2009). GLUT1 is widely distributed and responsible for basal level glucose uptake; GLUT3 is primarily expressed in neurons, but it is also found in other human cells, such as oligodendrocytes (Bell et al., 1990).

### Mitochondrial respiration (figure 1⑤, table 2)

We found no significant differences in the FCCP/protein ratio between controls (PBS-treated cells) and haloperidol-treated cells after 6 or 24 h. Thus, our data cannot associate haloperidol action to mitochondrial respiration in the applied experimental setting. Notably, these measures in an incubation chamber may not perfectly mirror haloperidol's effects on endogenous cell respiration in the culture dish. This is important, because an inhibition of mitochondrial respiration and free radical induction have been suggested as a mechanism of haloperidol neurotoxicity in the past (Arnaiz et al., 1999).

The clozapine-dependent decrease in extracellular glucose concentration may be due to stimulation of glycolysis. Elevated lactate concentrations measured after clozapine treatment suggest an increase in anaerobic glycolysis, which would lead to reduced ATP synthesis. However, the data of the current mitochondrial respiration analysis did not suggest a clozapine-induced decrease in the amount of mitochondria and the capacity of the respiratory chain for ATP synthesis (FCCP/protein ratio). Moreover, increased levels of the FCCP/oli ratio in the presence of clozapine indicated a lower degree of consumed oxygen molecules that were not coupled to ATP synthesis. In addition, the increase of the FCCP/oli ratio supports the possibility of clozapine-induced changes in the lipid composition of the mitochondrial membrane system. In fact, changes in lipid metabolism were evident at the level of FFA and galactocerebroside synthesis (see below). Taken together, these data suggest that the enhancement of glucose turnover ameliorates ATP synthesis via mitochondrial oxidative phosphorylation as well as anaerobic lactate formation.

With regard to lipid synthesis such an increased lactate generation results in elevated nicotinamide adenine dinucleotide phosphate (NADPH) levels that in turn support the synthesis of FFA and lipids. Additionally, changes in the permeability of the mitochondrial membrane system (leak respiration) may be linked to decreased thermogenesis. Such a lowered energy demand, mediated by the activity of the mitochondrial citric acid cycle, might again contribute to higher NADPH levels, thus supporting lipid synthesis.

### Myelin lipid synthesis (figures 1 ⑥–⑧, 4, 5)

Enhanced glycolysis and high lactate levels induced by clozapine are likely to trigger lipogenesis and myelin synthesis, since these processes require the availability of lactate in oligodendrocytes (Sanchez-Abarca et al., 2001).

Indeed, the present data suggest an increased expression of ACC1, the rate limiting key enzyme for FFA synthesis. Furthermore, clozapine but not haloperidol treatment was associated with an increased expression of galactocerebroside. Accordingly, the turnover of FFA in OLN-93 oligodendrocytes was increased by clozapine, leading to a decrease of their intracellular concentration. These data hint at a metabolic scenario which might explain why the administration of clozapine, by improving myelination and maintaining connectivity in the central nervous system, is associated with a superior longtime effect on psychotic symptoms. Studies in rodents have not yet systematically examined the influence of antipsychotic drugs on myelin lipid synthesis. However, previous results suggest that clozapine and quetiapine show superior effects regarding remyelination and oligodendrocyte maturation (in C57BL/6 mice suffering from cuprizone-induced white matter damage; Xiao et al., 2008; Xu et al., 2010; Zhang et al., 2012). Xu et al. observed less social interaction in mice given the myelin-toxic agent cuprizone for 28 days (Xu et al., 2010). Supporting the finding of a superior effect of clozapine on oligodendrocytes and myelin integrity, these behavioral changes were ameliorated by clozapine or quetiapine, but not by haloperidol (Xu et al., 2010).

### Limitations and potential questions

While cell culture studies do not necessarily reflect the *in vivo* pathophysiology and drug effects within the diseased brain, the present results indicate distinctly different actions of haloperidol and clozapine on of the energy metabolism and maturation of oligodendrocytic OLN-93 cells.

Our acute model nicely shows how these drugs act fast over the cellular proteome and metabolism. Antipsychotics' metabolic side-effects typically take a considerable amount of time to manifest in humans. Then it is natural to think that cellular effects are also delayed. Our results suggest earlier mechanisms. We defend that the delay in observing weight gain for example is due to the fact that there must be a systemic glycolytic dysfunction, which starts in the brain, but will only reach the hepatic cells after a while, if the antipsychotic treatment is continuous.

### Summary

Our results suggest that clozapine and haloperidol modulate differently oligodendrocytic glucose and lactate homeostasis, as well as myelin lipid synthesis. On the basis of clinical observations that antipsychotic drugs with the greatest clinical efficacy have the greatest metabolic effects, such as in the case of clozapine, it has been suggested that therapeutic and adverse effects of antipsychotic drugs (in particular clozapine) are related through influencing energy metabolism (Girgis et al., 2008). Our novel insights indicate that clozapine treatment might improve the energy supply and maturation of oligodendrocytes. Moreover, clozapine is apparently superior compared to haloperidol in maintaining the integrity of myelinated fibers. These findings support the concept that, in addition to rebalancing neurotransmission, certain antipsychotics may act as oligodendrocyte-modulators, improving neuronal connectivity.

The presented data suggest glycolysis as a central biochemical pathway underlying the effects of both antipsychotics on glucose and lactate availability in oligodendrocytes. While haloperidol treatment led to higher extracellular levels of glucose and lower intracellular levels of lactate, suggesting reduced glycolysis, clozapine improved glucose uptake as well as production and release of lactate.

Future studies should try to get a better understanding of these processes, e.g., by applying co-cultures with astrocytes and neurons or by using animal experiments.

Understanding the action of antipsychotic drugs in oligodendrocytes may help to develop novel cellular- or myelin-directed therapies for patients suffering from schizophrenia.

### Conflict of interest statement

The authors declare that the research was conducted in the absence of any commercial or financial relationships that could be construed as a potential conflict of interest.

## Footnotes

^1^ACC1 is the rate limiting key enzyme for FFA synthesis (catalytic formation of malonyl-CoA, a precursor for long chain fatty acyl-CoA, from acetyl-CoA). Myelin mainly consists of lipids (70–85%) and even though there are no absolutely “myelin-specific” lipids, galactocerebroside, also known as galactosylceramide, is the most typical one (Morell and Quarles, 1999).

## Abbreviations

HT1A: 5HT2A, serotonin receptors ACC1, acetyl-CoA carboxylase
ANOVA: analysis of variance
ATP: adenosine triphosphate
cDNA: complemetary deoxyribonucleic acid
CNP: 2′,3′-cyclic nucleotide 3′-phosphohydrolase
CO_2_: carbon dioxide
D2: D3, D4, dopamine receptors
DMEM: Dulbecco's modified Eagle's medium
DNA: deoxyribonucleic acid
EDTA: ethylenediaminetetraacetic acid
fc: fold change
FCCP: p-trifluoromethoxyphenylhydrazone
FFA: free fatty acids
GAPDH: glyceraldehyde-3-phosphate dehydrogenase
GLUT: glucose transporter
HC: high clozapine vs. control
HH: high haloperidol vs. control
LC: low clozapine vs. control
LH: low haloperidol vs. control
M4: acetylcholine receptor
MAG: myelin-associated glycoprotein
MCT: monocarboxylate transporter
MOG: myelin oligodendrocyte glycoprotein
NADPH: nicotinamide adenine dinucleotide phosphate
NG2: a transmembrane chondrotin sulfate proteoglycan expressed on immature glial progenitor cells
n.s.: not significant
oli: oligomycin
Olig1: Olig2, oligodendroglial transcription factors
OLN-93: an oligodendrocytic cell line from rat
PBS: phosphate buffered saline
PLP: proteolipid protein
RNA: ribonucleic acid
RPMI: Roswell Park Memorial Institute medium
RT-PCR: reverse transcriptase polymerase chain reaction
SCAP: sterol cleavage activation protein
s.e.m.: standard error of mean
SREBP: sterol regulatory element-binding protein
TBS-T: mixture of Tris-Buffered Saline and Tween 20
TREAT: effect of treatment
